# The Book World of Medicine and Science

**Published:** 1904-04-16

**Authors:** 


### The Book World of Medicine and Science.

A Manual of Operative Surgery. By Sir Frederick Treves, Bart., K.C.V.O., C.B., assisted by Jonathan Hutchinson, Jnr., F.R.C.S. New edition. Two volumes. With pp. 750 and 824, and illustrations 450 and 470 respectively. (London: Cassell and Company, Limited. 1903. Price 42s.)

When this work first appeared in 1891 it met a great want, and its pages were very widely perused. Since that date numerous treatises on operative surgery have appeared both in this country, on the Continent, and in America. Thus the present edition of the book has to compete with many and weighty rivals. There is somehow a feeling akin to disappointment on reviewing the pages as they now stand. Although there has been an attempt to bring the work up to the standard of the day, yet there is something that seems to be lacking. Whether this lies in the fact that there is an actual want of up-to-dateness, or in an absence of thoroughness and completeness, it is a little difficult to say. In connection with the former, it is only necessary to point out that there are illustrations of instruments which should have no place in the armamentarium of any surgeon, and their presence is without any note that they are altogether antiquated. Examples of this are to be seen in Figs. 420 and 431 in Vol. II., but there are many others, and they constitute grave blots upon the pages. In connection with the latter point it may be noted that there is no mention of any of the operative procedures that may be required for diseases of the pancreas, although such should find a place in all modern text-books. After having stated the above general impression, it becomes necessary to deal with the book more in detail, and to allude to some of the facts for which there cannot be other than praise. The first volume begins with general principles, and the account given of the patient, and the conditions militating against the success of operations could hardly be improved upon. There is some danger at the present day of teaching the student that for success the essentials must be as elaborate as they are found in a modern hospital, but in spite of this danger, the pages and illustrations apportioned to the description of a thoroughly well equipped operating theatre cannot but be of service. The difference in this chapter to that which appeared in the first edition is a striking example of the strides which have been made in recent years. It is interesting to note that Mr. Hutchinson has not hesitated to enter some valuable remarks of his own where his opinion is not in complete accord with Sir Frederick Treves, and this adds value to the pages. On the whole, the part dealing with the ligation of arteries is good, though there are a few blemishes, such as the want of an illustration of a good aneurism needle, although three altogether bad patterns are included. It is, moreover, unfortunate that the extraperitoneal method of ligaturing the common and internal iliac arteries is placed first, when it should have a subsidiary position to the transperitoneal operation. In the part dealing with operative measures upon the nerves there is a very clear account of the steps needed for excision of the Gasserian ganglion, and Mr. Hutchinson is thoroughly in favour of the operation and believes in its permanent benefit to the sufferer. Nothing is said as to the advantages of nerve anastomosis in certain cases of paralysis. The original author is particularly happy in his descriptions of amputations, and the pitfalls into which the operator may slip. Sir Frederick is much in favour of the subastragaloid disarticulation of the foot, and of the long external flap in amputations at the seat of election in the leg. Again the section on excisions is excellent, save for two figures, modified from Farabeuf, but not sufficiently modified for present day asepsis. It would have been desirable to have had some account of tendon implantation in that part dealing with tenotomy. The second volume commences with plastic surgery, and this is a most helpful section. In dealing with tuberculous glands in the neck, Sir Frederick does not advocate any of the heroic measures which are apt to be proposed at the present day, and possibly with reason on his side. Again there is little but praise for the sections on abdominal surgery, so far as general details are concerned, but there is some lack of fulness when individual operations are under review. The article on intestinal obstruction and the operative procedures necessary for its relief is very instructive and repays careful perusal. It is in connection with the relief of strangulated hernia that there would seem to be most to dissent from. On page 525 it is stated that the incision for an inguinal hernia should not encroach upon the tissue of the scrotum, and this is right, but the advice is entirely undone by the figure which is introduced on page 537, in which the incision does so, and only one half of the pubic hair is removed. Further the directions given concerning the division of the stricture at the neck of the sac, at least so far as an inguinal hernia is concerned, are not of the best. It should be taught that at the present day the stricture in such a case is divided from without inwards, and not from within outwards and altogether in the dark. Again the position indicated for the incision in a femoral hernia is wrong, and it is stated to be so in the text, and there can be no reason for the introduction of the illustration, save to confuse the reader. The desirability of completing if possible the operation for the relief of strangulation by the usual manipulations for radical cure is mentioned, but the discussion of this latter procedure is not given until the next section. It might have been well to have combined the two sections, or at least to have transposed them. Sir William Macewen's name is wrongly spelt in the text. The work on the whole, therefore, would have been somewhat more satisfactory if a little more care had been taken over its revision.

The Food of the Gods. A Popular Account of Cocoa. By Brandon Head. (London: R. Brimley Johnson, 4 Adam Street, Adelphi, W.C. 1903. Price 3s. 6d.)

This pleasantly-written and well-illustrated little book contains all that any reasonable person should want to know about the nature, dietetic value, and cultivation of cocoa, as well as a great deal of information concerning the plant which is curious and interesting, if not of special practical value. Among other things, it makes it clear that the British Empire might grow a great deal more cocoa for itself than it does at present. The district of Guayaquil, in the Republic of Ecuador, leads the world in the production of cacao, and has a larger output than the whole of Africa, which continent, however, is in many parts well adapted for its culture. Trinidad and Grenada produce a large amount for their size, but it is strange to find that, although these islands are British possessions, the owners of the cacao estates are nearly all French or Spanish by descent. Ceylon also produces some cacao, but the cultivation of the plant there does not seem to be increasing. The tree is also being cultivated in Samoa, and the author gives an interesting quotation from a letter by Robert Louis Stevenson telling how he helped to plant the precious beans in the neighbourhood of Vailima. A long chapter is devoted to a description of Messrs. Cadbury's village of cocoa makers at Bournville. Altogether this book is worth reading, even by those who have no particular interest in the growing and manufacturing of cocoa, although to these it will be specially interesting.

